# Regression of follicular lymphoma with Devil’s Claw: coincidence or causation?

**DOI:** 10.3747/co.v16i4.401

**Published:** 2009-08

**Authors:** K.S. Wilson

**Keywords:** Low-grade lymphoma, alternative therapy, cox-2 inhibition

## Abstract

**Background:**

Cancer patients frequently use alternative therapies. Two follicular lymphoma patients who had objective tumour regression after taking Devil’s Claw without cytotoxic therapy are reported here.

**Methods and Results:**

Patient 1 presented with coexistent immunoglobulin G plasma cell dyscrasia and stage iiia lymphoma (nodes 5 cm in diameter). Computed tomography scan 10 months later showed partial regression. On enquiry, it was learned that the patient was taking Devil’s Claw and Essiac (Essiac Products Services, Pompano Beach, FL, U.S.A.). This patient later developed overt myeloma, at which time he stopped the herbal supplements and underwent high-dose chemotherapy and stem cell transplantation, since which no lymphoma progression has occurred. Patient 2 presented with stage iiia lymphoma (nodes 2.5 cm in diameter). He learned of patient 1 through our lymphoma patient support group and started Devil’s Claw. Computed tomography scan 11 months later showed decreased adenopathy and splenomegaly, which has been sustained for 4 years.

**Discussion and Conclusions:**

Devil’s Claw tuberous root has anti-inflammatory properties, probably through suppression of cyclooxygenase 2 (cox-2) and inducible nitric oxide synthase expression. There are no prior reports of anticancer activity. Inhibition of cox-2 has a role in colon cancer prevention, has been implicated in lymphomagenesis, and is associated both with lymphoma stage and with response to treatment. However, spontaneous regression in lymphoma has been reported in 16% of patients in one series, of whom none were on herbal medications or cox-2 inhibitors. The key issue in both these patients is whether disease regression was “spontaneous” or causally related to therapy with Devil’s Claw. The timing of the response suggests a positive effect. Further investigation is warranted, preferably with a cox-2 inhibitor of known purity.

## 1. INTRODUCTION

Several clinical trials have investigated the role of “immediate” as opposed to “delayed” chemotherapy in asymptomatic patients with advanced low-grade lymphoma 1–6. Treatment intensity has varied from mild (oral chlorambucil) to multi-agent aggressive (ProMACE–CytaBOM). Immediate chemotherapy regimens have consistently failed to show a survival advantage over delayed treatment. Accordingly, patients are recommended to follow a surveillance program to monitor for symptoms and signs of progression that would warrant treatment intervention. More recently, new biologic treatment options, notably rituximab and tositumomab, which are effective both in untreated and in previously treated patients, have emerged, but remain untested in phase iii trials in asymptomatic patients 7–9.

In cancer patient populations, use of complementary and alternative therapies is widespread 10, reflecting the current state of the art with respect to conventional systemic therapies—that is, the failure of those therapies to cure most of the common metastatic epithelial malignancies and their well-known short- and long-term toxicity profiles. Even the “sensitive” cancers—most low-grade and a substantial proportion of intermediate-grade B-cell malignancies, most T-cell malignancies, and most adult leukemia— are often fundamentally incurable. In a study of 26 surveys from 13 countries, the use of complementary and alternative therapies ranged from 7% to 64%. In adult patients, the average prevalence of such use was 31% 11. Here, I report two patients who used Devil’s Claw supplements and who had objective tumour regressions confirmed on computed tomography (ct) scanning. Whether these regressions were causally related to the intervention or were coincidental is uncertain, but the timing of the intervention points to a treatment effect.

## 2. PATIENTS AND METHODS

From a personal malignant lymphoma practice during 28 years at the BC Cancer Agency, I report two patients with low-grade lymphoma manifesting disease regression after the use of Devil’s Claw supplements.

Patient 1 presented at age 57 in January 2000 with a left lower cervical neck lymph node mass 5 cm in diameter. Incisional biopsy revealed follicular lymphoma, grade 2. He was asymptomatic. Physical examination was otherwise normal. An abdominal ct scan showed a left para-aortic lymph node mass 5 cm in diameter. Bone marrow examination showed abnormal plasma-cell infiltrate (5% of cellularity) with kappa monoclonality. Relevant investigations included serum M protein (10.4 g/L); serum immunoglobulin G (IgG) kappa, IgA and IgM (normal); albumin (40 g/L); calcium (2.25 mmol/L); creatinine (87 μmol/L); β^2^-microglobulin (1.1 mg/L); and skeletal survey (negative).

**FIGURE 1:**
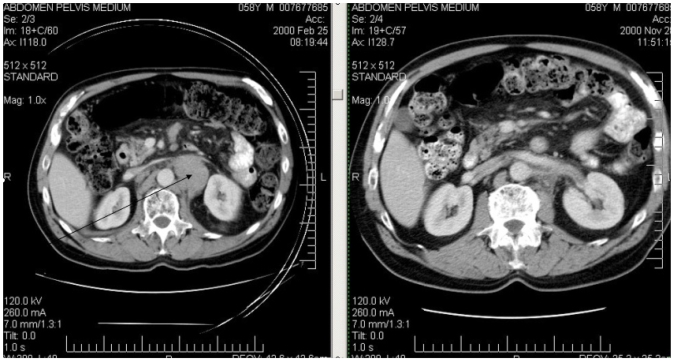
Patient 1: abdominal computed tomography scans showing regression of retroperitoneal adenopathy.

Because this patient was asymptomatic, observation was recommended, with review in 3 months. Of his own volition, he started taking supplements of Devil’s Claw extract 500 mg daily and Essiac (Essiac Products Services, Pompano Beach, FL, U.S.A.) from a health food store. When assessed in July 2000, the left neck mass was no longer palpable. Follow-up ct scan in November 2000 showed a marked reduction in the size of the retroperitoneal nodes (Figure 1). He developed overt myeloma in August 2001, at which time he stopped the herbal supplements and received dexamethasone, followed by high-dose melphalan, cyclophosphamide, and autologous peripheral blood stem-cell rescue. His myeloma responded, but there has since been evidence of paraprotein progression. No clinical progression of his cervical adenopathy has been observed through September 2008, probably as a result of the high-dose chemotherapy. He has not had a further ct scan because of contrast risk in patients with myeloma proteins.

**FIGURE 2:**
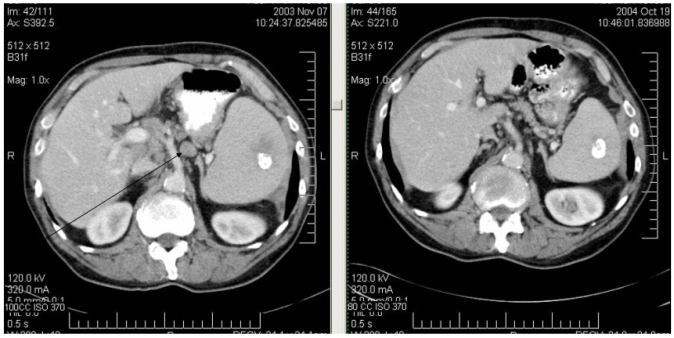
Patient 2: computed tomography scans showing decreased adenopathy and splenomegaly.

Patient 2 presented in November 2003 at age 69 with transient abdominal pain, which led to ultrasound examination. Imaging showed splenomegaly and retroperitoneal adenopathy. There were no constitutional symptoms. On physical examination, he had multiple enlarged superficial lymph nodes (neck, axillae, and inguinal regions) up to 2.5 cm in diameter. A ct scan confirmed widespread adenopathy and splenomegaly. There was also a calcified nodule in the spleen. Inguinal lymph node biopsy showed follicular lymphoma grade 1. With clinical stage iiia disease, observation was recommended. He learned of patient 1 through our lymphoma patient support group and started Devil’s Claw supplements 500 mg daily. Follow-up abdominal ct 11 months later showed “significant interval improvement” in adenopathy and splenomegaly, which has been sustained through April 2007 (Figure 2). In September 2008, only 1 cervical lymph node (1 cm in diameter) was palpable. He continues to take Devil’s Claw supplements.

## 3. DISCUSSION AND SUMMARY

Devil’s Claw (*Harpagophytum procumbens* 12) tuberous root contains harpagoside and β-sitosterol. It has anti-inflammatory and analgesic properties, probably through suppression of cyclooxygenase-2 (cox-2) and inducible nitric oxide synthase expression 13. There is evidence supporting its use in osteoarthritis and low back pain 14, but none indicating anticancer activity. Inhibition of cox-2 has an accepted role in cancer prevention 15, has been implicated in lymphomagenesis 16, and is associated both with stage of lymphoma and with response to treatment 17. Expression of cox-2 has been reported in myeloma 18, but use of Devil’s Claw in patient 1 did not prevent myeloma evolution.

Patient 1 also took Essiac 19,20, a herbal tea with several constituents, including burdock root, Indian rhubarb root, sheep sorrel, and slippery elm. The only positive anticancer effect observed in preclinical testing was in prostate and ovarian cancer cell lines; all leukemia, lymphoma, sarcoma, and solid-tumour tests were negative 19,21. No formal phase i or ii clinical trials of Essiac have been conducted. A retrospective chart review of 86 Canadian patients who took Essiac could not attribute changes in cancer status to Essiac. Accordingly, the lymphoma regression in patient 1 is likely not attributable to Essiac.

The key issue in both these patients is whether the disease regression was “spontaneous” or causally related to Devil’s Claw therapy. Neither patient was taking any other herbal or prescription medications besides those described. In low-grade lymphoma, apparently-spontaneous regression has been reported in 7 of 44 patients (16%) on observation only 22 and has lasted from 6 to 60+ months (median: 14 months). In 2 of those patients, a viral illness occurred before the observed lymphoma regression, but no patients were known to be on herbal medications or cox inhibitors. Altogether, a total of 9 patients experienced spontaneous regression, including 2 patients with aggressive lymphomas, for a total incidence of 20%. In another series, spontaneous regressions were more frequent in follicular than in diffuse lymphoma (13% vs. 3%, chi-square *p* = 0.02), and 7 of 20 spontaneous remissions lasted more than 1 year 23.

Potential explanations for spontaneous regressions have been reviewed 24. Infections may stimulate immune surveillance by cellular and antibody-mediated mechanisms. The role of immune surveillance is exemplified by the development of lymphoma, mostly higher grade, in organ transplant patients receiving immunosuppressive therapy. However, most lymphoma patients exhibiting spontaneous regression do not have a precipitating factor. In the cases reported here, the only “intervention” or “event” was the use of Devil’s Claw supplements. The chance of observing spontaneous regression in 2 consecutive lymphoma patients can be estimated at approximately 2%.

To evaluate the anticancer effects of Devil’s Claw, further laboratory and clinical testing would be necessary. However, Devil’s Claw has not been evaluated by Health Canada or the U.S. Food and Drug Administration for safety or purity. Furthermore, no regulated manufacturing standards are currently in place for complementary medicines. Indeed, some herbal and health supplements have been found to be contaminated with toxic metals or other drugs. Considering the role of cox-2 inhibitors and the relationship between the components of Devil’s Claw and cox-2, it may be more prudent to investigate an established cox-2 inhibitor in low-grade lymphoma, given that the manufacture, chemical constituents, and toxicity profiles of those agents are well known.
